# A cross-sectional study on the correlation between fasting blood glucose and bone turnover markers in Chinese patients with osteoporotic fractures

**DOI:** 10.3389/fmed.2025.1564957

**Published:** 2025-04-10

**Authors:** Meng-cheng Zhu, Min-zhe Xu, Chang-xuan Li, Jia-hao Wang, Chong Li, Ya-qin Gong, Jian Jin, Ke Lu, Yan-ming Hao

**Affiliations:** ^1^Department of Orthopedics, Affiliated Kunshan Hospital of Jiangsu University, Suzhou, Jiangsu, China; ^2^Kunshan Biomedical Big Data Innovation Application Laboratory, Suzhou, Jiangsu, China; ^3^Department of Orthopedics, The First People's Hospital of Kunshan, Gusu School, Nanjing Medical University, Suzhou, Jiangsu, China; ^4^Information Department, Affiliated Kunshan Hospital of Jiangsu University, Suzhou, Jiangsu, China; ^5^Kunshan Municipal Health and Family Planning Information Center, Suzhou, Jiangsu, China

**Keywords:** fasting blood glucose, procollagen type 1 N-terminal propeptide, β-C-terminal telopeptide of type I collagen, bone turnover markers, osteoporosis, osteoporotic fractures

## Abstract

**Background:**

Recent studies suggest that metabolic factors, such as fasting blood glucose (FBG), may significantly affect bone health, influencing the risk and severity of osteoporotic fractures (OPFs). This study examined the association between FBG levels and bone turnover markers (BTMs) in patients hospitalized for OPFs requiring surgical intervention.

**Methods:**

A retrospective cross-sectional analysis was conducted on 888 patients treated for OPFs at Kunshan Hospital affiliated with Jiangsu University from November 2018 to August 2023. Serum levels of FBG, procollagen type 1 N-terminal propeptide (P1NP), and β-C-terminal telopeptide of type I collagen (β-CTX) were measured, with FBG serving as an independent variable, and P1NP and β-CTX as outcome variables. Patients were stratified into tertiles based on FBG levels, and multiple regression models were adjusted for confounding variables, including age, gender, BMI, and clinical parameters. Non-linear relationships and threshold effects were analyzed.

**Results:**

Adjusted regression models identified a negative association between FBG and BTMs. For each 1 mmol/L increase in FBG, β-CTX levels decreased by 0.02 ng/mL (95% CI: −0.04 to −0.01; *p* < 0.01), and P1NP levels decreased by 2.91 ng/mL (95% CI: −4.38 to −1.45; *p* < 0.01). Non-linear relationships were observed, with an inflection point at 7.93 mmol/L for both markers. Below this threshold, higher FBG levels were associated with a steeper decline in BTMs.

**Conclusion:**

FBG levels exhibit a negative non-linear association with P1NP and β-CTX in patients with OPFs. Elevated FBG levels may adversely affect BTMs, potentially contributing to the progression of osteoporosis (OP). These findings underscore the importance of glycemic control in managing bone health among patients with OPFs.

## 1 Introduction

Osteoporosis (OP) is a prevalent bone disease marked by decreased bone mineral density (BMD) and thinning of bone tissue, resulting in a higher susceptibility to fractures (1). Fractures resulting from OP constitute a significant source of mortality and disability among the elderly population (2, 3). Globally, more than 1/3 of women and 1/5 of men over 50 are at risk of sustaining an osteoporosis-associated fracture in their lifetime (4). With aging populations and rising life expectancies, the global cost of treating osteoporotic fractures (OPFs) is expected to increase, posing significant challenges to public health and healthcare systems (5). Identifying factors contributing to OP and OPFs is crucial for improving bone health and mitigating associated socioeconomic burdens.

Fasting blood glucose (FBG) is widely used to monitor glycemic control in diabetes and is associated with risks of cardiovascular disease, chronic kidney disease, fractures, and infections (6). Recent research highlights a complex association between FBG levels and bone health across different populations and health conditions. For example, research conducted on non-diabetic elderly women has demonstrated a positive association between elevated FBG levels and increased BMD, while lower glucose levels have been linked to a higher risk of OP (7). Another research indicates that in the Korean population, higher FBG levels are inversely associated with OP risk, particularly in individuals with impaired fasting glucose (IFG) or diabetes (8). Furthermore, findings by Kim et al. highlight that greater variability in FBG significantly raises the risk of overall and spinal fractures in the Korean non-diabetic population, underscoring the importance of maintaining stable glucose levels for bone health (9).

During the bone remodeling process, bone turnover markers (BTMs) are released and can be quantified in blood or urine samples, providing an indication of the bone remodeling rate (10). Changes in bone strength cannot be detected using Dual-energy X-ray absorptiometry (DXA) scans; therefore, BMD measured by DXA does not fully represent compromised bone quality (11). Therefore, BTMs may serve as alternatives to BMD measurements for monitoring the effectiveness of OP treatment (12). Specific BTMs, such as procollagen type 1 N-terminal propeptide (P1NP) and β-C-terminal telopeptide of type I collagen (β-CTX), are highly sensitive and specific serological indicators. These markers are recommended by the International Federation of Clinical Chemistry and Laboratory Medicine (IFCC) and the International Osteoporosis Foundation (IOF) for assessing bone turnover levels (13). P1NP, a precursor of type I collagen synthesized by osteoblasts, enters the bloodstream during bone formation, whereas β-CTX is secreted by osteoclasts during bone resorption (14). Moreover, serum BTMs can rapidly reflect metabolic changes in bone formation and resorption, aiding in the identification of individuals at high risk for fragility fractures (15).

Patients with type 2 diabetes mellitus (T2DM) and normal BMD often exhibit an elevated fracture risk, which is typically associated with impaired bone quality rather than reduced bone quantity (16, 17). This indicates that evaluating the association between FBG and bone quality, as measured by BTMs, may be more effective in identifying individuals at high risk for fragility fractures. Although prior studies have explored the relationship between FBG and BTMs in populations such as T2DM. (18), children aged 9 to 11 (19), and individuals with cystic fibrosis-related diabetes (CFRD) (20), this relationship remains unexplored in patients with OPFs undergoing surgical intervention. Addressing this research gap is essential to understanding the metabolic factors influencing bone remodeling in this population.

## 2 Materials and methods

### 2.1 Study design and subjects

**Figure 1:**
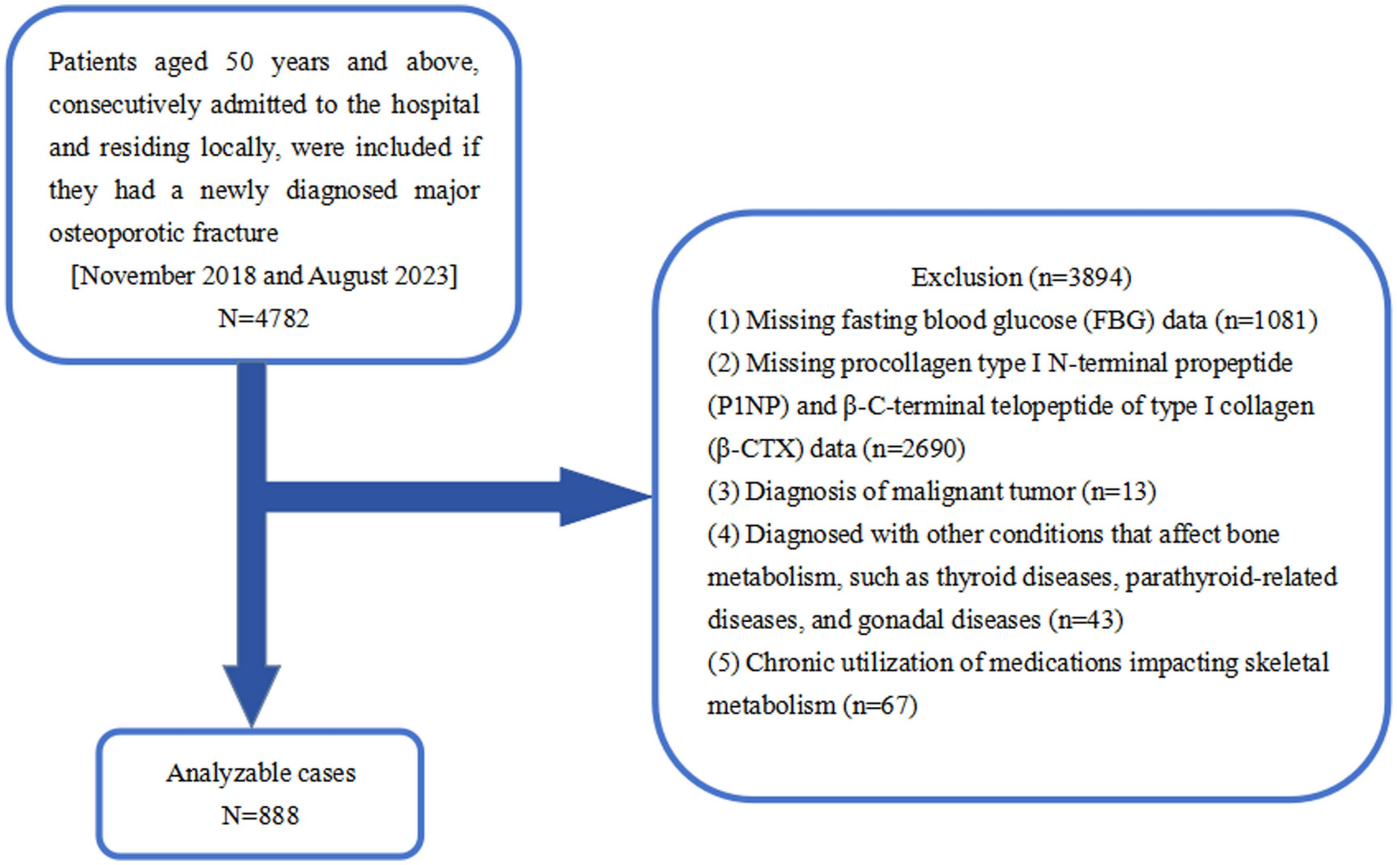
Study flowchart.

This cross-sectional retrospective study encompassed 4,782 individuals with OPFs who were admitted to Kunshan Hospital of Jiangsu University and underwent surgical intervention between November 2018 and August 2023. OP is characterized by fragility fractures that can occur even with normal BMD T-scores and in the absence of other metabolic bone diseases; a diagnosis of OP can be confirmed with a T-score of ≤ −2.5, without visible fractures, at specific sites including the femoral neck, lumbar spine, distal third of the forearm, and total hip (21). The exclusion criteria included: (1) Missing FBG data (*n* = 1,081); (2) Missing data for P1NP and β-CTX (*n* = 2,690); (3) Diagnosed malignant tumors (*n* = 13); (4) Disorders impacting bone metabolism, such as thyroid, parathyroid, or gonadal abnormalities (*n* = 43); and (5) Long-term use of bone metabolism-affecting medications (*n* = 67). Finally, a total of 888 patients were eligible for the current analysis (Figure 1).

### 2.2 Exposure and outcome variables

FBG levels were measured using the hexokinase method with a Beckman AU5800 automated biochemistry analyzer, and these values served as independent variables. All FBG measurements were conducted after at least 8 h of fasting to ensure accuracy. Outcome variables included P1NP and β-CTX, measured using the electrochemiluminescence immunoassay (ECLIA) method with a Roche Cobas 8,000 analyzer. All procedures were performed by certified laboratory technicians following standardized protocols and calibrated instrumentation.

### 2.3 Covariate variables

The covariates included age, gender, BMI, albumin, aspartate aminotransferase (AST), serum creatinine (CR), blood urea nitrogen (BUN), alanine aminotransferase (ALT), hemoglobin, hypertension, and the American Society of Anesthesiologists (ASA) score. The ASA classification, determined by anesthesiologists during preoperative evaluations, reflects the severity of a patient’s underlying conditions and their potential impact on anesthesia management (22). All laboratory parameters were measured from blood samples collected after an 8-h fast. ALT and AST were quantified using enzymatic colorimetric methods, while BUN and CR were measured enzymatically with a Beckman AU5800 biochemical analyzer. BMI was calculated as weight (kg)/height^2^ (m^2^). All laboratory measurements followed standardized operating procedures (SOPs) and were conducted by certified laboratory technicians.

### 2.4 Statistical analysis

Categorical variables were assessed using Pearson’s chi-square test or Fisher’s exact test, while continuous data were examined using independent t-tests or Mann–Whitney U tests, based on the distribution normality. Univariate analyses were conducted to assess the associations between BTMs and patient characteristics in individuals undergoing surgical interventions for OPFs. Generalized Estimating Equations (GEE) is a statistical modeling approach for correlated data that estimates the parameters of a generalized linear model while taking into account potential correlations, and is particularly applicable to longitudinal or clustered datasets. The present study used GEE for statistical analyses. In the absence of distinct clustering, GEE results are essentially equivalent to those of ordinary linear regression; the use of the approach in this study was motivated primarily by the robustness of parameter estimation that GEE provides. GEE was used to adjust for covariates and examine independent associations between FBG levels and BTMs. Three models were used for analysis: Model 1 was unadjusted; Model 2 was adjusted for age, gender, and BMI; Model 3 incorporated additional adjustments beyond Model 2, including ALT, AST, BUN, CR, Albumin, ASA score, hypertension, and hemoglobin. Multicollinearity was assessed using Variance Inflation Factor (VIF) analysis with a threshold of ≥10. All covariates in the final model showed acceptable VIF values, specifically, age (1.1), gender (1.1), BMI (1.0), ALT (3.2), AST (3.1), BUN (1.4), CR (1.4), albumin (1.3), hemoglobin (1.3), ASA score (1.0), and hypertension (1.1), indicating no significant multicollinearity concerns. Covariates were included in the models if they met the following criteria: (1) a change in standardized regression coefficients (β) or odds ratios (OR) ≥10% upon inclusion or exclusion; and (2) a *p-*value ≤ 0.1 in univariate analyses or Model 1. Potential non-linear relationships were identified using Generalized Additive Models (GAM). Piecewise linear regression models were applied to determine threshold effects for significant non-linear associations. Segmented regression analysis with recursive methods and maximum likelihood estimation was used to calculate breakpoints where differing slope ratios were observed. Statistical significance was set at a two-sided *p*-value < 0.05. Analyses were performed using the R statistical software (The R Foundation) and Empower Stats (X&Y Solutions Inc., Massachusetts, USA).

## 3 Results

### 3.1 Patient characteristics

Table 1 summarizes the baseline characteristics of 888 hospitalized patients with OPFs enrolled from November 2018 to August 2023, stratified by FBG tertiles (2.95–5.12, 5.13–5.98, and 5.99–19.11 mmol/L). The cohort consisted of 262 males (29.50%) and 626 females (70.50%), with a mean age of 69.41 ± 11.18 years. As described in the methods, all BTMs (P1NP and β-CTX) were measured from blood samples collected after an 8-h fast on the morning following admission, typically within 24–48 h of fracture occurrence. Mean serum levels of P1NP and β-CTX were 57.75 ± 34.85 ng/mL and 0.56 ± 0.30 ng/mL, respectively, while mean FBG was 6.03 ± 1.82 mmol/L. A significant decline in β-CTX levels was observed across increasing FBG tertiles (T1: 0.62 ± 0.33 ng/mL; T2: 0.56 ± 0.28 ng/mL; T3: 0.49 ± 0.26 ng/mL; *p* < 0.01). Similarly, P1NP levels showed a marked reduction with higher FBG tertiles (T1: 65.26 ± 32.79 ng/mL; T2: 57.21 ± 30.79 ng/mL; T3: 50.83 ± 39.05 ng/mL; *p* < 0.01).

**Table 1:** Profile of participants in the study.

FBG, mmol/L	Total	T1 (2.95–5.12)	T2 (5.13–5.98)	T3 (5.99–19.11)		
	Mean ± SD	Mean ± SD	Mean ± SD	Mean ± SD	*p*-value	*p*-value^*^
N	888	294	298	296		
Age, year	69.41 ± 11.18	70.24 ± 11.59	68.73 ± 11.19	69.26 ± 10.74	0.25	0.26
BMI, kg/m^2^	23.52 ± 3.37	23.54 ± 3.18	23.51 ± 3.44	23.50 ± 3.50	0.99	0.95
ALT, U/L	22.11 ± 16.41	21.14 ± 18.95	22.16 ± 15.18	23.02 ± 14.80	0.38	<0.01
AST, U/L	24.71 ± 15.10	23.99 ± 17.19	24.92 ± 14.50	25.22 ± 13.41	0.59	0.24
β-CTX, ng/ml	0.56 ± 0.30	0.62 ± 0.33	0.56 ± 0.28	0.49 ± 0.26	<0.01	<0.01
P1NP, ng/ml	57.75 ± 34.85	65.26 ± 32.79	57.21 ± 30.79	50.83 ± 39.05	<0.01	<0.01
BUN, mmol/L	6.17 ± 2.17	6.13 ± 1.79	5.94 ± 2.23	6.45 ± 2.42	0.02	<0.01
CR, μmoI/L	63.09 ± 24.70	61.23 ± 18.40	64.94 ± 29.71	63.08 ± 24.55	0.19	0.41
Albumin, g/L	39.75 ± 3.67	39.64 ± 3.75	39.87 ± 3.53	39.75 ± 3.73	0.76	0.77
Hemoglobin, g/L	127.27 ± 15.64	127.47 ± 15.87	128.06 ± 14.99	126.28 ± 16.03	0.37	0.48
Gender, *N* (%)					0.11	–
Female	626 (70.50%)	211 (71.77%)	197 (66.11%)	218 (73.65%)		
Male	262 (29.50%)	83 (28.23%)	101 (33.89%)	78 (26.35%)		
Hypertension, *N* (%)					0.80	–
No	757 (85.25%)	251 (85.37%)	251 (84.23%)	255 (86.15%)		
Yes	131 (14.75%)	43 (14.63%)	47 (15.77%)	41 (13.85%)		
ASA score, *N* (%)					0.55	–
1	136 (15.32%)	49 (16.67%)	47 (15.77%)	40 (13.51%)		
≥2	752 (84.68%)	245 (83.33%)	251 (84.23%)	256 (86.49%)		

*p*-value*: Kruskal Wallis Rank Test for continuous variables, Fisher Exact for categorical variables with Expects < 10.

SD, standard deviation; FBG, fasting blood glucose; T1, first tertile; T2, second tertile; T3, third tertile; BMI, body mass index; ALT, alanine aminotransferase; AST, aspartate aminotransferase; β-CTX, beta-C-terminal telopeptide of type I collagen; P1NP, procollagen type I N-terminal propeptide; BUN, blood urea nitrogen; CR, creatinine; ASA, American Society of Anesthesiologists.

### 3.2 Univariate analyses of factors associated with BTMs

Univariate analysis revealed significant correlations between CR, hemoglobin, and albumin with P1NP levels, with no significant associations found among other variables. For β-CTX, significant associations were identified with ALT, AST, CR, albumin, gender, and hemoglobin. No significant relationships were observed for other covariates (Table 2).

**Table 2:** Univariate analysis for BTMs.

	Statistics^a^	P1NP β^b^ (95%CI) *p*-value	β-CTX β^b^ (95%CI) *p*-value
Age, year	69.41 ± 11.18	**−0.0016 (−0.2068, 0.2035)** 0.99	**−0.0013 (−0.0030, 0.0005)** 0.15
BMI, kg/m^2^	23.52 ± 3.37	−0.23 (−1.00, 0.54) 0.56	**−0.0025 (−0.0087, 0.0037)** 0.42
ALT, U/L	22.11 ± 16.41	−0.08 (−0.22, 0.05) 0.23	**−0.0019 (−0.0031, −0.0007)** <0.01
AST, U/L	24.71 ± 15.10	0.01 (−0.14, 0.16) 0.87	**−0.0021 (−0.0034, −0.0008)** <0.01
BUN, mmol/L	6.17 ± 2.17	0.50 (−0.55, 1.56) 0.35	**0.0082 (−0.0008, 0.0172)** 0.07
CR, μmoI/L	63.09 ± 24.70	0.15 (0.05, 0.24) <0.01	**0.0014 (0.0006, 0.0022) <0.01**
Albumin, g/L	39.75 ± 3.67	−1.16 (−1.80, −0.52) <0.01	**−0.0087 (−0.0141, −0.0033)** <0.01
Hemoglobin, g/L	127.27 ± 15.64	−0.33 (−0.47, −0.18) <0.01	**−0.0015 (−0.0027, −0.0002)** 0.02
FBG, mmol/L	6.03 ± 1.82	−3.10 (−4.35,-1.86) <0.01	−0.03 (−0.04,-0.02) <0.01
Gender, *N* (%)			
Female	626 (70.50%)	Reference	Reference
Male	262 (29.50%)	3.96 (−1.06, 8.98) 0.12	0.07 (0.02, 0.11) <0.01
Hypertension, *N* (%)			
No	757 (85.25%)	Reference	Reference
Yes	131 (14.75%)	0.07 (−6.40, 6.54) 0.98	**−0.0001 (−0.0553, 0.0550)** 1.00
ASA score, *N* (%)			
1	136 (15.32%)	Reference	Reference
≥2	752 (84.68%)	−1.64 (−8.01, 4.73) 0.61	−0.05 (−0.10, 0.01) 0.09

The data highlighted in bold now exhibit four decimal places, thereby improving the sensitivity and precision of the dataset.

^aFor continuous variables.^

^bThe dependent variable was BTMs and β is the result of univariate analysis for BTMs.^

CI, confidence interval; BTMs, bone turnover markers; β-CTX, beta-C-terminal telopeptide of type I collagen; P1NP, procollagen type I N-terminal propeptide; BMI, body mass index; ALT, alanine aminotransferase; AST, aspartate aminotransferase; BUN, blood urea nitrogen; CR, creatinine; FBG, fasting blood glucose; ASA, American Society of Anesthesiologists.

### 3.3 FBG and BTMs associations

Tables 3, 4 present the relationships among FBG, P1NP, and β-CTX. In the unadjusted Model 1, significant negative associations were identified between FBG and P1NP (β = −3.10, 95% CI: −4.35 to −1.86, *p* < 0.01) and between FBG and β-CTX (β = −0.03, 95% CI: −0.04 to −0.02, *p* < 0.01). In Model 2, which was adjusted for basic demographic factors, these associations remained significant (P1NP: β = −2.91, 95% CI: −4.34 to −1.48, *p* < 0.01; β-CTX: β = −0.02, 95% CI = −0.04 to −0.01, *p* < 0.01). The fully adjusted Model 3 confirmed the significance of the associations between FBG and P1NP (β = −2.91, 95% CI: −4.38 to −1.45, *p* < 0.01) and between FBG and β-CTX (β = −0.02, 95% CI: −0.04 to −0.01, *p* < 0.01).

**Table 3:** Relationship between FBG and P1NP across various models.

	Model 1^a^ *N* = 888		Model 2^b^ *N* = 749		Model 3^c^ *N* = 727	
	β (95%CI)	*p*-value	β (95%CI)	*p*-value	β (95%CI)	*p*-value
FBG, mmol/L	−3.10 (−4.35, −1.86)	<0.01	−2.91 (−4.34, −1.48)	<0.01	−2.91 (−4.38, −1.45)	<0.01
Tertile FBG, mmol/L						
T1 (2.95–5.12)	Reference		Reference		Reference	
T2 (5.13–5.98)	−8.05 (−13.59, −2.51)	<0.01	−8.80 (−15.04, −2.56)	0.01	−9.45 (−15.78, −3.11)	<0.01
T3 (5.99–19.11)	−14.43 (−19.98, −8.88)	<0.01	−14.35 (−20.76, −7.94)	<0.01	−14.74 (−21.25, −8.24)	<0.01

^aAdjust for none.^

^bAdjust for age, gender, BMI.^

^cAdjust for age, gender, BMI, ALT, AST, BUN, CR, Albumin, hemoglobin, hypertension, ASA.^

CI, confidence interval; FBG, fasting blood glucose; T1, first tertile; T2, second tertile; T3, third tertile; P1NP, procollagen type I N-terminal propeptide; BMI, body mass index; ALT, alanine aminotransferase; AST, aspartate aminotransferase; BUN, blood urea nitrogen; CR, creatinine; ASA, American Society of Anesthesiologists.

**Table 4:** Relationship between FBG and β-CTX across various models.

	Model 1^a^ *N* = 888		Model 2^b^ *N* = 749		Model 3^c^ *N* = 727	
	β (95%CI)	*p*-value	β (95%CI)	*p*-value	β (95%CI)	*p*-value
FBG, mmol/L	−0.03 (−0.04, −0.02)	<0.01	−0.02 (−0.04, −0.01)	<0.01	−0.02 (−0.04, −0.01)	<0.01
Tertile FBG, mmol/L						
T1 (2.95–5.12)	Reference		Reference		Reference	
T2 (5.13–5.98)	−0.07 (−0.11, −0.02)	0.01	−0.07 (−0.12, −0.02)	0.01	−0.06 (−0.11, −0.01)	0.02
T3 (5.99–19.11)	−0.13 (−0.18, −0.08)	<0.01	−0.13 (−0.18, −0.08)	<0.01	−0.13 (−0.18, −0.08)	<0.01

^aAdjust for none.^

^bAdjust for age, gender, BMI.^

^cAdjust for age, gender, BMI, ALT, AST, BUN, CR, Albumin, hemoglobin, hypertension, ASA.^

CI, confidence interval; FBG, fasting blood glucose; T1, first tertile; T2, second tertile; T3, third tertile; β-CTX, beta-C-terminal telopeptide of type I collagen; BMI, body mass index; ALT, alanine aminotransferase; AST, aspartate aminotransferase; BUN, blood urea nitrogen; CR, creatinine; ASA, American Society of Anesthesiologists.

In the tertile analysis, the levels of both P1NP and β-CTX showed a clear stepwise decline as the FBG tertiles increased. For P1NP, the most pronounced reduction occurred in T3, where the levels decreased by approximately 14.35–14.74 ng/mL across all three models compared to the reference tertile (T1) (all *p* < 0.01). T2 showed more modest reductions in P1NP, ranging from 8.05 to 9.45 ng/mL across the three models (all *p* ≤ 0.01). Similarly, for β-CTX, patients in T3 showed a consistent reduction of 0.13 ng/mL across all models compared to T1 (all *p* < 0.01). Smaller reductions of 0.06–0.07 ng/mL (all *p* ≤ 0.02) were found in T2.

### 3.4 Spline smoothing plot and threshold analyses

**Figure 2:**
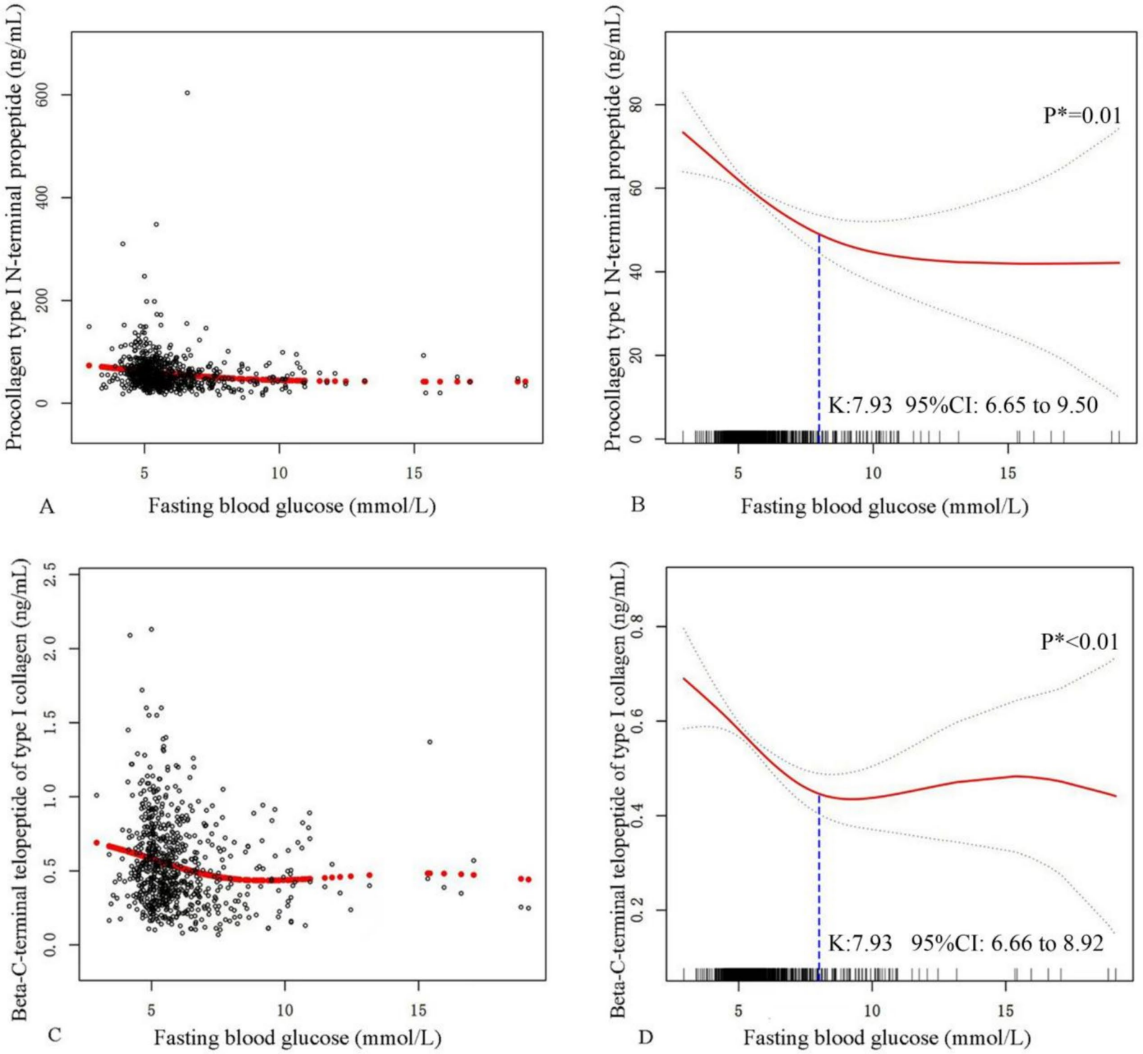
Smoothed curve analysis with adjustments was conducted to elucidate the relationship between FBG and BTMs. **(A,C)** Every black dot signifies an individual sample from a participant, depicting the relationship between FBG and P1NP in panel **(A)** and between FBG and β-CTX in panel **(C)**. **(B,D)** Red curve lines represent the smooth curve fits between FBG and P1NP in panel **(B)** and between FBG and β-CTX in panel **(D)**, with dashed line representing the 95% confidence intervals from the fits. Models were adjusted for Age, gender, BMI, ALT, AST, BUN, CR, albumin, hemoglobin, hypertension, and ASA. Abbreviations: FBG, fasting blood glucose; BTMs, bone turnover markers; β-CTX, beta-C-terminal telopeptide of type I collagen; P1NP, procollagen type I N-terminal propeptide; BMI, body mass index; ALT, alanine aminotransferase; AST, aspartate aminotransferase; BUN, blood urea nitrogen; CR, creatinine; ASA, American Society of Anesthesiologists. * *p*-values calculated by the Likelihood Ratio Test (LRT).

The analysis revealed distinct non-linear relationships between FBG levels and BTMs (β-CTX and P1NP) in patients with OPFs (Figure 2). For both β-CTX and P1NP, an identical inflection point was identified at an FBG level of 7.93 mmol/L, which was used as the threshold for further correlation analyses. Below the inflection point of 7.93 mmol/L, FBG demonstrated a significant negative correlation with both β-CTX and P1NP. The effect size for FBG and P1NP below this threshold was −6.02 (95% CI: −8.76 to −3.28, *p* < 0.01), while the effect size for FBG and β-CTX was −0.06 (95% CI: −0.08 to −0.04, *p* < 0.01). However, no significant associations were observed between FBG and either marker above the threshold. The effect size for FBG and P1NP above 7.93 mmol/L was 0.32 (95% CI: −2.50 to 3.14, *p* = 0.82), while for FBG and β-CTX, the effect size was 0.01 (95% CI: −0.01 to 0.03, *p* = 0.36) (Table 5).

**Table 5:** Analysis of thresholds to explore the Association between FBG and BTMs.

	Model 3^a^	
	P1NP β (95% CI) *p*-value	β-CTX β (95% CI) *p*-value
Model A^b^		
One line slope	−2.91 (−4.38, −1.45) <0.01	−0.02 (−0.04, −0.01) <0.01
Model B^c^		
FBG turning point (K), mmol/L	7.93	7.93
95% CI for K	6.65, 9.50	6.66, 8.92
<K	−6.02 (−8.76, −3.28) <0.01	−0.06 (−0.08, −0.04) <0.01
>K	0.32 (−2.50, 3.14) 0.82	0.01 (−0.01, 0.03) 0.36
Slope 2-Slope 1	6.34 (1.61, 11.07) 0.01	0.07 (0.03, 0.10) <0.01
P1NP value at K, ng/mL or β-CTX value at K, ng/mL	45.64 (38.79, 52.50)	0.42 (0.37, 0.48)
LRT^d^	0.01	<0.01

^aAdjusted for age, gender, BMI, ALT, AST, BUN, CR, Albumin, hemoglobin, hypertension, ASA.^

^bLinear analysis, p-value < 0.05 indicates a linear relationship.^

^cNonlinear analysis.^

^dp-value < 0.05 indicates that Model B differs significantly from Model A, which indicates a nonlinear relationship.^

Abbreviations: CI, confidence interval; FBG, fasting blood glucose; BTMs, bone turnover markers; β-CTX, beta-C-terminal telopeptide of type I collagen; P1NP, procollagen type I N-terminal propeptide; BMI, body mass index; ALT, alanine aminotransferase; AST, aspartate aminotransferase; BUN, blood urea nitrogen; CR, creatinine; ASA, American Society of Anesthesiologists.

## 4 Discussion

OPFs, predominantly resulting from low BMD and deteriorated bone microarchitecture, represent a significant health challenge among the elderly population (23). These fractures significantly impair the quality of life and independence. Findings from this study demonstrate that elevated FBG levels are inversely associated with BTMs, specifically β-CTX and P1NP. For each one-unit increase in FBG, β-CTX levels decrease by 0.02 ng/mL (95% CI: −0.04 to −0.01; *p* < 0.01), while P1NP levels decline by 2.91 ng/mL (95% CI: −4.38 to −1.45; *p* < 0.01). A non-linear correlation between FBG and BTMs was established, with a threshold concentration of 7.93 mmol/L observed.

Previous research has focused on the association between blood glucose and BMD. A Mendelian randomization study suggested that FBG may reduce hip bone area in non-diabetic populations while potentially increasing BMD (24). Similarly, a cross-sectional study reported a positive correlation between FBG and BMD in non-diabetic elderly Chinese women, indicating that lower FBG levels may increase the risk of OP in this group (25). BMD represents the static accumulation of bone mineral content, and thus represents an effective quantification of bone mass. However, BMD measurements have inherent limitations, as they are unable to provide a comprehensive analysis of bone microarchitecture and qualitative properties (26). While DXA is effective in assessing the mineral density and content of osseous tissue, it cannot evaluate biochemical activities and dynamic remodeling processes (27). In contrast, BTMs, such as P1NP and β-CTX, provide a direct reflection of the dynamic biochemical processes involved in bone metabolism. These markers provide real-time assessments of matrix renewal and skeletal remodeling, thus capturing dynamic changes in bone quality that remain undetected by measures of static density (27).

The findings of this study are generally consistent with previous research on the relationship between blood glucose levels and BTMs. For example, in a study conducted at Aarhus University Hospital in Denmark, researchers found that in patients with T2DM, β-CTX levels were negatively correlated with both the amplitude of glycemic excursions and FBG levels, whereas P1NP showed a significant negative correlation only with FBG (18). Similarly, a cross-sectional study from Denmark found lower P1NP and β-CTX levels in patients with CFRD compared to those without cystic fibrosis (20). The underlying mechanisms for these associations remain to be fully elucidated. Insulin resistance (IR), often associated with IFG and impaired glucose tolerance (IGT), is considered a key factor. In individuals with abnormal glucose levels, reduced BTMs are associated with increased IR and compromised pancreatic β-cell function (28). The trend of reduced β-CTX levels is particularly evident in adolescents with IFG, as shown by research from Nicolaus Copernicus University in Poland, with more pronounced effects in boys, while the impact on P1NP appeared to be minimal in this group (19). Furthermore, studies report that individuals with IFG and diabetes in Australian populations exhibit significantly lower levels of P1NP and β-CTX compared to those with normal glucose levels (29). Research involving glucose tolerance tests further highlights the complex interplay between glucose metabolism and BTMs. For example, intravenous glucose tolerance tests have shown that β-CTX and P1NP reach their lowest levels at specific time points in individuals with normal glucose tolerance (30). Similarly, oral glucose tolerance tests in patients with hyperthyroidism, β-thalassemia, and healthy individuals indicate suppression of β-CTX levels, though P1NP levels remain unaffected (31). The consistent decrease in BTMs across various glucose dysregulation conditions highlights glucose metabolism’s pivotal role in bone health. Differential effects on β-CTX and P1NP indicate distinct regulatory mechanisms. Further research is needed to clarify these mechanisms and develop interventions to mitigate their adverse impacts on skeletal health.

Bones function not only as structural supports but also as endocrine organs, influencing a wide range of tissues and systems, including muscles, blood vessels, the immune system, the brain, kidneys, the liver, and the pancreas (32). Osteocalcin, a hormone secreted by osteoblasts, plays a pivotal role in glucose metabolism by enhancing insulin secretion and sensitivity, thereby contributing to lower blood glucose levels (32, 33). Bone metabolism and glucose metabolism are interdependent, forming a complex, symbioticrelationship.

This study systematically investigated the relationship between FBG levels and BTMs in patients with OPFs undergoing surgical interventions while accounting for multiple confounding factors. A significant inverse association between FBG and BTMs was identified, characterized by a distinct non-linear relationship and a threshold effect.

Physiological concentrations of insulin promote osteoblast proliferation and inhibit the proliferation and differentiation of osteoclasts (34). Nevertheless, IR disturbs this balance by hindering osteoblast proliferation and stimulating osteoclast activation through suppression of the Wnt/β-catenin signaling pathway (35, 36). Although IR reduces bone turnover rates and may increase bone density, it simultaneously enhances bone fragility by increasing cortical porosity within the bone microarchitecture (34).

The impact of hyperglycemia on bone metabolism is complex, encompassing various mechanisms that collectively lead to decreased bone density and elevated risk of OP. Earlier research has demonstrated that hyperinsulinemia and the buildup of advanced glycation end-products (AGEs) suppress BTMs (37). Chronic hyperglycemia promotes the production of AGEs, which impair protein structure and function through increased non-enzymatic collagen cross-linking (38). AGEs bind to their specific receptor, upregulating transforming growth factor-beta (TGF-β) expression and secretion, which suppresses osteoblast differentiation and mineralization while enhancing receptor activator of nuclear factor kappa-B ligand (RANKL) expression, thereby stimulating osteoclastogenesis and disrupting bone mineralization (39–41). Moreover, elevated glucose and AGE levels enhance sclerostin expression, inhibiting the Wnt signaling pathway and suppressing bone formation (42, 43). High glucose levels induce reactive oxygen species production in osteoblasts, exacerbating oxidative stress that impairs Wnt/β-catenin signaling, promotes apoptosis in osteoblasts and osteocytes, and increases adipocyte formation, thereby exacerbating bone loss (44, 45). Furthermore, hyperglycemia induces a pro-inflammatory microenvironment characterized by elevated levels of systemic markers such as IL-6 and TNF-*α*, which correlate with disrupted bone turnover and increased skeletal fragility through mechanisms distinct from direct glucose-mediated effects (46). While our findings suggest a potential link between elevated blood glucose and reduced bone turnover, the exact mechanisms remain speculative. Further studies are warranted to explore the roles of AGEs, inflammation, IR, and Wnt/β-catenin signaling in this association.

This study’s findings have significant clinical implications. The observed declines in P1NP and β-CTX (2.91 ng/mL and 0.02 ng/mL per 1 mmol/L increase in FBG, respectively) highlight suppressed bone turnover processes. This suppression may impair bone remodeling and compromise bone quality, increasing the risk of skeletal fragility over time. Based on the recommendations of the American Diabetes Association (ADA), diabetes mellitus is diagnosed when FBG levels reach ≥ 7.0 mmol/L, while values between 5.6 and 6.9 mmol/L indicate prediabetes or IFG (47). The threshold observed in the present study (7.93 mmol/L), which is slightly higher than that of the diagnostic criteria for diabetes, has significant clinical implications. This threshold likely represents the physiological influence of glucose on bone metabolism: when FBG remains below this threshold, BTM levels decrease linearly as the glucose levels rise; however, once FBG exceeds this threshold, the inhibitory effect may plateau or be counteracted by compensatory mechanisms, resulting in a flattened curve. These findings suggest that sustained mild hyperglycemia can already affect bone turnover and reduce the capacity for bone tissue renewal and repair, without requiring the development of severe hyperglycemia. Evidence suggests that poor glycemic control is associated with a higher risk of fractures in patients with T2DM, although this relationship has not been definitively established (48). Additionally, separate research has demonstrated that BTM levels are reduced in patients with poor glycemic control compared to those with good control or no T2DM (49). Our findings support the implementation of routine hyperglycemia screening in older patients with low BMD or fracture history, advocating for stricter monitoring and control of FBG levels through interdisciplinary collaborative interventions, particularly in patients with OPF, as a comprehensive strategy to improve bone quality and optimize treatment outcomes.

This study demonstrates several significant strengths while acknowledging certain limitations that must be addressed. By employing three statistical models, the study investigated the relationships between FBG, BTMs, and multiple potential confounding variables, offering valuable insights representative of the study population. The use of robust statistical methods and adjustments for confounding factors enhances the reliability of the findings. However, the primary limitation of this cross-sectional study is its inability to establish causal relationships. The analysis, based on data from a single time point, cannot clarify whether elevated FBG levels directly lead to a decrease in BTMs or are instead a consequence of other underlying factors. Moreover, as all data were sourced from a single institution, Kunshan Hospital, affiliated with Jiangsu University, the generalizability of the results to broader or diverse populations may be limited. Despite adjustments for numerous confounding factors, this study has several limitations in terms of unmeasured variables that could potentially influence the association between FBG and BTMs. Specifically, these unmeasured variables encompass lifestyle factors (smoking, alcohol consumption, diet, exercise), as well as other health-related parameters (vitamin D status, HbA1c, medications for diabetes). The use of single time-point measurements of FBG and BTMs represents an additional limitation, as these biomarkers fluctuate naturally and may be influenced by acute fracture stress. Our exclusion of patients with missing BTMs data is also a limitation, potentially introducing selection bias. It is likely that the clinicians ordered BTMs selectively for patients with more severe or atypical presentations, which limits the generalizability of our findings. These constraints emphasize the necessity of carrying out prospective longitudinal research to gain a deeper understanding of the temporal dynamics of the interaction between FBG and BTMs.

## 5 Conclusion

In summary, this study demonstrates a negative and non-linear relationship between FBG and BTMs, specifically P1NP and β-CTX, in patients with OPFs. The findings suggest that higher FBG levels may impair bone turnover, potentially increasing the risk of OP. These results highlight the potential utility of FBG as a biomarker for assessing the prognosis and risk of OPFs. Further research is warranted to elucidate the mechanistic underpinnings of the relationship between FBG and bone metabolism and to validate the prognostic value of FBG in a larger and more diverse patient population. Given the cross-sectional design of the study, it was not possible to establish a causal relationship between hyperglycemia and reduced bone turnover rate; rather, we can only demonstrate an association between these variables.

## Data Availability

The original contributions presented in the study are included in the article/supplementary material, further inquiries can be directed to the corresponding authors.
